# Practical Guide to Bone Marrow Sampling for Suspected Myelodysplastic Syndromes

**Published:** 2017-01-01

**Authors:** Jean A. Ridgeway, Sara Tinsley, Sandra E. Kurtin

**Affiliations:** 1 University of Chicago Medical Center, Chicago, Illinois;; 2 H. Lee Moffitt Cancer Center and Research Institute, Tampa, Florida;; 3 University of Arizona Cancer Center, Tucson, Arizona;

## Abstract

Myelodysplastic syndromes (MDS) comprise a group of diverse clonal hematopoietic stem cell malignancies that are characterized by ineffective hematopoiesis and progressive bone marrow failure. Clinical symptoms are generally nonspecific. The diagnosis, classification, and risk stratification of MDS rely on the evaluation of peripheral blood and bone marrow sampling using the Revised International Prognostic Scoring System tool. Accurate diagnosis and risk stratification require a good-quality bone marrow sample. Bone marrow samples are obtained using two complementary techniques: bone marrow aspiration and bone marrow biopsy. Knowledge of what constitutes an adequate bone marrow sample and a proper bone marrow sampling technique may help advanced practitioners obtain quality samples while minimizing patient discomfort and risk. Patient preparation and positioning, site selection, sampling equipment, and sampling technique can help lead to the collection of high-quality bone marrow samples. Postprocedural care and knowledge of potential complications can reduce a patient’s pain and optimize recovery

Characterized by ineffective hematopoiesis and progressive bone marrow failure, myelodysplastic syndromes (MDS) comprise a group of diverse clonal hematopoietic stem cell malignancies (Brunning et al., 2008; Zeidan, Faltas, Douglas Smith, & Gore, 2013). Deficiencies in hematopoiesis result in peripheral blood cytopenias, of which anemia is the most common. Thrombocytopenia and neutropenia, although less common, can lead to significant morbidity and mortality. The risk of progression to acute myeloid leukemia (AML) is linked to a range of clinical features and cytogenetic abnormalities (Cazzola, 2011).

Evidence suggests that MDS may be more common than previously thought: At a conservative estimate, approximately 60,000 individuals are living with MDS in the United States, and more than 10,000 new cases are diagnosed annually (Ma, 2012; Ma, Does, Raza, & Mayne, 2007). The incidence of MDS increases markedly with age. Given the aging population in the United States, the number of cases of MDS is expected to escalate in the coming years, with an associated increase in disease morbidity (Ma, 2012). The leading cause of death in patients with MDS remains the disease itself.

Myelodysplastic syndromes are among the most challenging of the myeloid neoplasms to diagnose and classify (Vardiman et al., 2009). Clinical symptoms are generally nonspecific, and the comprehensive diagnosis, classification, and risk stratification of the disease rely on a series of investigations of peripheral blood and bone marrow samples that assess cytogenetic changes in hematopoietic cells and cell lineages (Lee, Erber, Porwit, Tomonaga, & Peterson, 2008).

Bone marrow samples are obtained using two complementary techniques: bone marrow aspiration and bone marrow biopsy; both types of sample are generally required for an accurate diagnosis (Lee et al., 2008). The use of appropriate technique during the performance of these procedures is critical not only to obtain sufficient, correctly prepared, high-quality bone marrow samples to facilitate diagnosis and classification, but also to minimize patient discomfort and reduce procedure-associated side effects. This article provides an overview of the importance of bone marrow examination for patients with suspected MDS, including its pivotal role in diagnosis, classification, and risk stratification.

## DIAGNOSIS AND CLASSIFICATION OF MDS

Diagnosing MDS can be challenging, as many of the clinical features, such as anemia, are nonspecific. In fact, many patients may be asymptomatic, and MDS may only be suspected following an abnormal routine blood test (Cazzola & Malcovati, 2005). Therefore, MDS are primarily diagnosed and characterized by abnormal cell morphology (dysplasia) and quantitative changes in one or more blood and bone marrow cell lines; it is not possible to diagnose MDS without having both blood and bone marrow samples.

There are a number of MDS classification systems, including that of the World Health Organization (WHO; Vardiman et al., 2009). According to the WHO classification criteria, MDS can be classified into seven major subgroups based on information obtained from peripheral blood and bone marrow (Vardiman et al., 2009). Bone marrow specimens are examined for the presence, proportion, and lineage of blasts; the degree and type of dysplasia; the presence of specific abnormal cellular morphology and inclusions; and specific chromosomal abnormalities (Vardiman et al., 2009).

Patients with MDS have a high rate of a variety of clonal chromosomal abnormalities. The most common cytogenetic abnormalities are incorporated into MDS risk stratification systems. First developed in 1997 by Greenberg and colleagues (1997), the International Prognostic Scoring System (IPSS; Table 1) is a commonly used tool to estimate overall survival and progression to AML in patients with newly diagnosed MDS. It stratifies patients into one of four risk categories based on the evaluation of the presence of cytopenias (including neutropenia, thrombocytopenia, and anemia) in the peripheral blood as well as the presence of blasts and cytogenetic abnormalities in bone marrow cells. In 2012, the IPSS was updated and refined, resulting in the Revised IPSS (IPSS-R); this tool reflects the increased knowledge of prognostic indicators and updates of other classification and prognostic systems (Table 2; Greenberg et al., 2012). The IPSS-R classifies patients using five cytogenetic categories, further division of bone marrow blast percentage groups, and depth of each cytopenia.

**Table 1:**
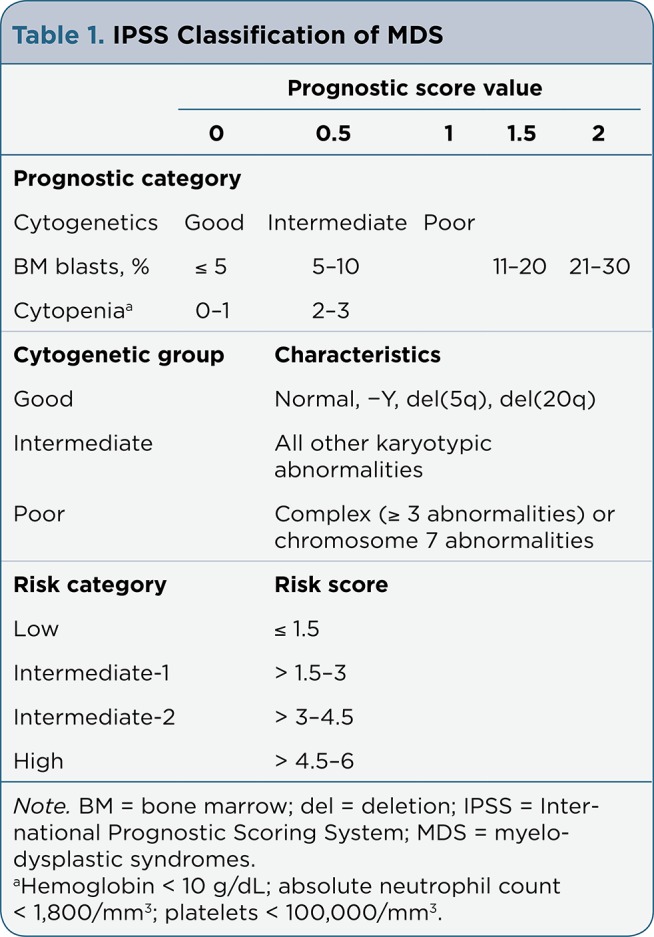
IPSS Classification of MDS

**Table 2:**
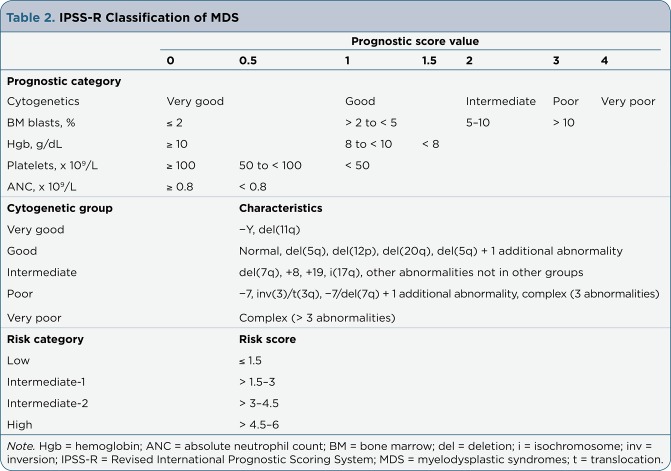
IPSS-R Classification of MDS

The different risk groups identified using the IPSS and IPSS-R are associated with significantly different median overall survival and time to AML progression. Therefore, IPSS-R risk stratification guides treatment selection. The IPSS-R is now the current standard for prognostic staging. Web-based calculators for the IPSS-R are now available for health-care practitioners, such as that on the MDS Foundation website (http://www.mds-foundation.org/ipss-r-calculator/).

**Case Study 1**

A 73-year-old woman presented to her primary care physician with progressive fatigue and shortness of breath. She was found to have macrocytic anemia and was subsequently referred to a hematologist. A complete blood cell count was performed: hemoglobin, 7.7 g/dL; absolute neutrophil count (ANC), 2.0 × 109/L; and platelet count, 266 × 109/L. A bone marrow biopsy was performed, showing normocellular bone marrow with 3% blasts and dysplasia that did not meet diagnostic criteria. Cytogenetics revealed del(5q)(13q35) in 20 of 20 metaphases. Based on the IPSS-R risk calculator, this patient’s score was 3.5, putting her in the intermediate-risk category (Table 3).

**Table 3:**
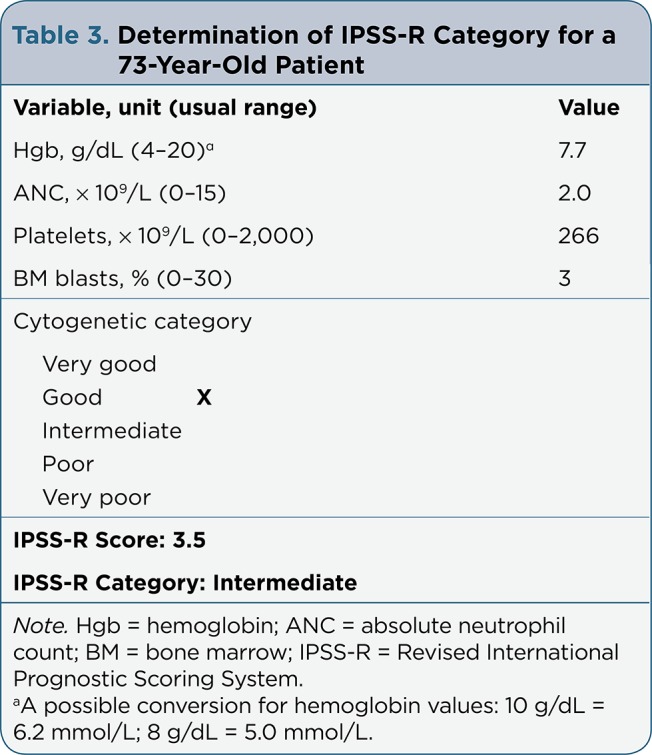
Determination of IPSS-R Category for a 73-Year-Old Patient

**Case Study 2**

A 62-year-old man had sequential infections over the winter. A complete blood cell count was performed: hemoglobin, 8.9 g/dL; ANC, 0.4 × 109/L; and platelet count, 108 × 109/L. The results of a bone marrow biopsy were 10% blasts and multilineage dysplasia, with a single repetitive clone (t 6:9) in 19 of 20 metaphases. Based on the IPSS-R risk calculator, this patient’s score was 5.5, putting him in the high-risk category Table 4).

**Table 4:**
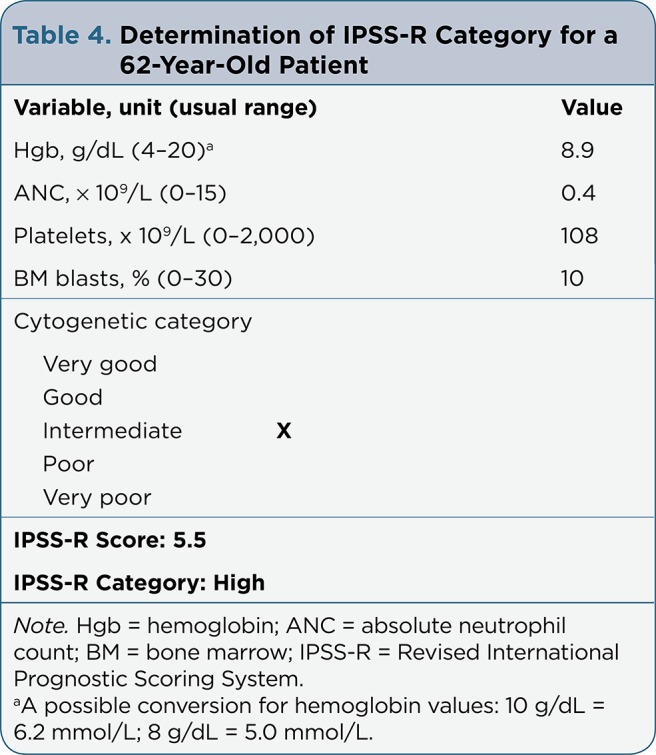
Determination of IPSS-R Category for a 62-Year-Old Patient

More recently, molecular profiling of patients with MDS has shown predictive and prognostic value and will likely be added to a future updated risk stratification of MDS (National Comprehensive Cancer Network, 2016). Molecular testing is performed on bone marrow aspirates.

## BONE MARROW SAMPLING TECHNIQUES

Bone marrow can be investigated using two complementary techniques: bone marrow aspiration and bone marrow biopsy (often called bone marrow trephine biopsy or needle-core biopsy). Bone marrow aspiration is the removal by suction of liquid bone marrow, whereas bone marrow biopsy involves the removal of an intact bone marrow sample. Aspiration and biopsy provide useful, and complementary, information. Obtaining both samples is generally required for an accurate diagnosis (Lee et al., 2008).

**Figure 1:**
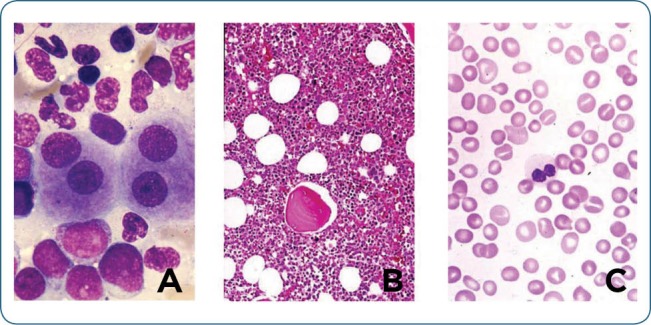
Cell morphology. (A) Bone marrow blasts and dysplasia. (B) Microstructure of the marrow, with stromal elements and hematopoietic cells. (C) Peripheral blood smear to assess abnormal cellular morphology, blast counts, and cytopenias.

**Figure 2:**
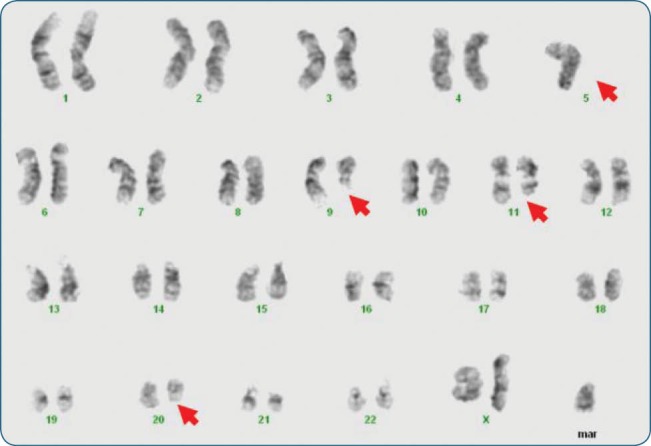
Metaphase cytogenetic analysis, with red arrows indicating clonal chromosomal abnormalities in a patient with MDS.

Bone marrow aspirates are used to evaluate cell morphology and perform a cell count of different marrow elements, including blasts (Figure 1A), whereas sections of a bone marrow biopsy allow the pathologist to visualize the microstructure of the marrow and describe overall cellularity (e.g., the ratio of hematopoietic tissue to fat), stromal elements (e.g., connective tissue), and the proportion and maturation of hematopoietic cells (Figure 1B). In addition to bone marrow samples, peripheral blood smears are also examined to assess abnormal cellular morphology, blast counts, and cytopenias (Figure 1C). Finally, cytogenetic analyses are performed on aspirate samples, or core samples if needed, using classical metaphase techniques and, in some cases, more specialized techniques such as fluorescence in situ hybridization to identify chromosomal abnormalities (Nybakken & Bagg, 2014). An example of a metaphase cytogenetic analysis to identify clonal chromosomal abnormalities in a patient with MDS is shown in Figure 2.

For these techniques to be performed successfully, and to avoid the need to subject the patient to repeated procedures, it is essential that adequate samples be obtained.

**What Constitutes an Adequate Bone Marrow Sample?**

**Figure 3:**
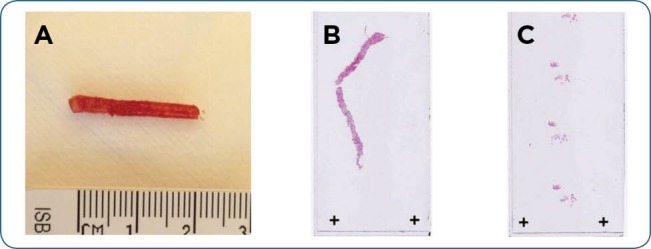
Bone marrow aspirate. (A) Sample size of at least 1.5 to 2 cm to allow for at least 10 partially preserved intertrabecular areas. (B, C) Ready touch prints of small biopsy sample.

The volume of bone marrow aspirate necessary will depend on the requirements specified by local laboratories. Bone marrow aspirates should be examined immediately for the presence of spicules; these small fragments of bone indicate that the marrow cavity has been accessed and that the aspiration has been performed correctly. Spicules are easily detected by examining a drop of the aspirate spread on a slide or dish. In the case of a bone marrow biopsy, a sample size of at least 1.5 to 2 cm is needed to allow for at least ten partially preserved intertrabecular areas (Figure 3; Lee et al., 2008; Vardiman et al., 2009).

Adequate bone marrow biopsy specimens are required to ensure that a representative sample is obtained to increase the accuracy of the evaluation. For example, in aggressive MDS, there may be aggregates (3–5 cells) or clusters (> 5 cells) of blasts, usually localized to the central portion of the bone marrow, away from the vascular structure and surfaces of the bone trabeculae (Brunning et al., 2008). If a poor sampling technique is used, they may be missed. If a bone marrow aspirate cannot be obtained, e.g., due to fibrosis or cellular packing, touch preparations of the bone marrow biopsy (which can be obtained from the same iliac crest) can be made by gently touching the unfixed core sample onto a slide. Two core biopsies are generally recommended. These slides are fixed and stained and can be used in place of aspirate samples (Lee et al., 2008; Vardiman et al., 2009).

## PRACTICAL CONSIDERATIONS

**Prebiopsy Assessment and Preparation**

Prior to the procedure, patients should be evaluated for the presence of any potential risk factors, such as the use of anticoagulants or any allergies to local anesthetics. The need for adequate antianxiety medication, sedation, and analgesia should also be assessed (Lee et al., 2008). Bone marrow aspiration and biopsy are painful procedures for the majority of patients (Hjortholm, Jaddini, Hałaburda, & Snarski, 2013).

One of the most important factors associated with procedural pain is patient anxiety (Hjortholm et al., 2013), and the degree to which pain is perceived during biopsy has been linked to the inadequate provision of information prior to the procedure (Degen, Christen, Rovo, & Gratwohl, 2010). It is, therefore, critically important for patients to be fully and accurately informed about what the process entails to reduce anxiety and prepare them for the procedure.

Intravenous sedation with drugs such as lorazepam or diazepam may be required to reduce anxiety in some patients, and it has the advantage of producing a retrograde amnesia following the procedure in many patients (Hjortholm et al., 2013). Sedation is often requested by patients who have experienced high levels of pain during a previous biopsy, highlighting the need to keep initial procedures as pain-free as possible (Hjortholm et al., 2013). However, the use of intravenous sedation has drawbacks in terms of the length of clinic stay, the need for additional staff and equipment, and an increased risk of adverse effects (Hjortholm et al., 2013).

**Site Selection and Patient Position**

**Figure 4:**
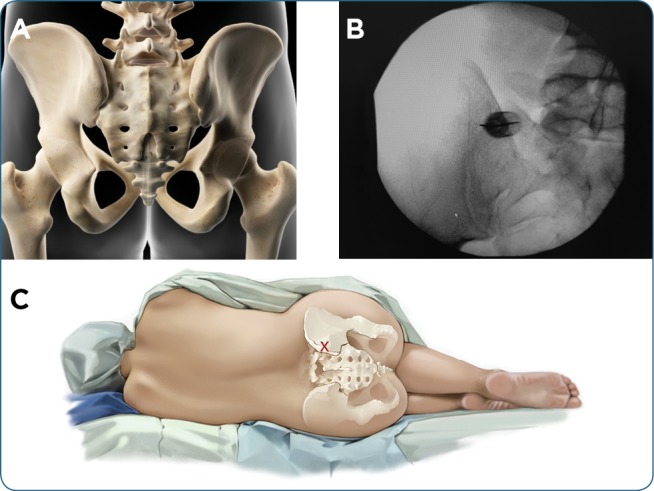
Site selection and patient position. (A) The posterior iliac crest is the preferred site for bone marrow aspiration and biopsy in adults. (B) Fluoroscopic-guided biopsy showing accurate placement of the needle. (C) Patient positioned in the left lateral decubitus position.

The posterior iliac crest is the preferred site for bone marrow aspiration and biopsy in adults (Figure 4A); the site is chosen because there are no major nerves in the area, if the correct anatomic area is located (Islam, 2013; Lee et al., 2008). If the patient is immobile or the posterior iliac crest is inaccessible due to obesity or injury, the anterior iliac crest can be used. The sternum is only suitable for aspirate samples and may be used in certain circumstances, e.g., in immobile patients from whom it has not been possible to obtain a sample from other areas or when trephine biopsy is not required (Lee et al., 2008). However, because of the risk of cardiac tamponade, sternal aspiration should only be performed by an experienced practitioner (Lee et al., 2008).

Ultrasonography and fluoroscopy can be useful when identifying the biopsy site; both are particularly useful when the patient is obese (Figure 4B; Islam, 2013). In addition, ultrasonography- and fluoroscopy-guided biopsies have been shown to allow highly accurate placement of the needle, avoiding any damage to neighboring structures (Islam, 2013; Möller et al., 2001).

For a posterior iliac crest biopsy, the patient is placed either in a prone position or in the right/left lateral decubitus position, with the knees flexed at 45° (Figure 4C). For anterior iliac crest or sternal biopsies, the patient is placed in a supine position. Patients should be made comfortable with pillows as required. The biopsy site is identified by careful palpation of anatomic landmarks or by using imaging as described previously, and the area is cleaned using an appropriate antiseptic solution (e.g., chlorhexidine or povidone-iodine solution) and draped.

The skin is first infiltrated slowly with local anesthetic (1%–2% buffered lidocaine in nonsensitive patients). Once the skin is numb, the subcutaneous tissue and periosteum are infiltrated with 2 to 5 mL of lidocaine, depending on the thickness of the subcutaneous tissue and the depth of the periosteum. Sufficient time (3–5 minutes) is given for the full anesthetic effects to develop. The adequacy of anesthesia can be tested by gently probing the periosteum with the tip of a needle and questioning the patient about any pain.

During the procedure, provision of information is again important; patients should be kept informed throughout and forewarned about potential discomfort, e.g., during local anesthetic administration and during sampling.

**Bone Marrow Sampling Equipment**

**Figure 5:**
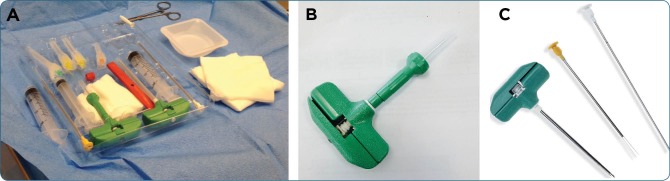
(A) Bone marrow procedure kit. (B) An "Illinois-type" aspiration needle, with sternal guard in place and a T handle. (C) "Trap-type" biopsy needles that "trap" the bone marrow specimen into the cannula with a concave cutting point.

An example of a bone marrow procedure kit is shown in Figure 5A. These kits provide a convenient combination of sterile instruments that will be used for the procedure. Custom kits can be ordered by individual offices/hospitals and usually include items for preparing and draping the patient, local anesthetic, syringes, and aspiration and biopsy needles. Sampling typically uses two needles at two adjacent sites in the bone, generally through the same incision (Islam, 2007; Lee et al., 2008; Malempati, Joshi, Lai, Braner, & Tegtmeyer, 2009).

With this method, aspirate samples are usually obtained using an Illinois needle, whereas a biopsy requires a trap, Jamshidi, Islam, or snare-coil needle. The two different types of needle are supplied by numerous companies throughout the United States, including Ranfac Corporation (http://www.ranfac.com), SOMATEX Medical Technologies GmbH (https://www.somatex.com), CareFusion (http://www.carefusion.com), and Argon Medical Devices (http://www.argonmedical.com). Figure 5B presents an example of an "Illinois-type" aspiration needle (shown with the sternal guard in place), with a T handle, which offers the practitioner stability while performing the procedure.Figure 5C shows an example of a "trap-type" biopsy needle that "traps" the bone marrow specimen into the needle cannula, using a concave cutting point.

A single-needle technique can also be used, which may reduce patient discomfort. The single-needle method uses specialized biopsy/aspiration needles (e.g., the Jamshidi bone marrow biopsy and aspiration needle). The biopsy needle is inserted for the first part of the procedure (aspiration), the marrow sample is removed using a syringe, and the needle is subsequently advanced to an additional depth of approximately 1.0 cm to obtain a core biopsy (Al-Ibraheemi et al., 2013). Although this method has been shown to provide adequate samples in some studies (Al-Ibraheemi et al., 2013), the two-needle method has had superior results in other studies (Islam, 2007).

An alternative to manual bone marrow biopsy equipment is the battery-powered Arrow OnControl Powered Bone Marrow Biopsy System (http://www.arrowoncontrol.com/), which was approved for use in the United States in 2007. This device can be used to obtain both aspirate and core biopsy samples and has been shown to provide longer core samples in a significantly shorter time (Miller et al., 2011). However, there is evidence to suggest that despite the larger biopsy sample obtained, the quality of the sample may be lower and with less evaluable marrow than in samples obtained manually (Lynch, Stauffer, & Rosenthal, 2015).

## SAMPLING PROCEDURE USING THE TWO-NEEDLE METHOD

The two bone marrow sampling procedures, aspiration and biopsy, may be performed in any order. Although aspiration is usually performed first, some practitioners recommend that it may be preferable to perform the biopsy first because aspiration is the more painful procedure (Dayton et al., 2014). However, if the biopsy is performed first, it can produce a hemodilute specimen or induce a hypercoagulable state and possible premature clotting of the aspirate (Islam, 2007). Alternatively, if the biopsy site is too close to the aspiration site, intramedullary hemorrhage in the biopsy can be seen (Islam, 2007).

**Aspiration**

To provide adequate negative pressure, it is recommended that the aspirate be drawn using a plain 10- or 20-mL plastic syringe attached to the aspiration needle (Lee et al., 2008).

To perform bone marrow aspiration, practitioners should:

1. Make an incision at the puncture site with a scalpel blade. If the bone marrow biopsy was performed first, use the same incision but adjust the angle slightly so the aspiration needle enters the bone at a different position, 0.5 to 1 cm away from the biopsy needle (Lee et al., 2008; Malempati et al., 2009).
2. Using the Illinois needle, remove the shaft guard from the aspiration needle, if present, to allow for adequate depth of penetration and check that the stylet can be easily removed from the cannula.
3. For optimal control and stability, hold the aspiration needle with the proximal end in the palm and the index finger against the side of the needle shaft near the tip.
4. Introduce the needle through the incision and bring the needle into contact with the periosteum. The patient should feel only a sensation of pressure at this point; if pain is experienced, either reposition the needle gently until the anesthetized area is found or administer more lidocaine.
5. Using gentle but firm pressure, advance the needle while rotating it in an alternating semicircular clockwise–counterclockwise motion. Once the needle passes through the cortical bone and enters the marrow cavity, there is generally a decrease in resistance, and the needle should stay in place without being held.
6. Remove the stylet from the needle, warn the patient that her or she may feel pain when bone marrow is being aspirated, and aspirate approximately 0.5 to 1 mL of bone marrow into the syringe.
7. Evaluate the aspirate immediately by spreading a drop of the sample on a slide or dish to observe bony spicules, which will be visible as irregularities in the smooth surface of the blood.
8. If spicules are not present, it may be necessary to repeat the procedure by inserting the needle further into the bone or repeat the procedure at a new site (Malempati et al., 2009).
9. Prepare samples immediately following aspiration; initial smears should be made with aspirate directly from the plain collecting syringe; remaining aspirate should be placed into tubes containing ethylenediaminetetraacetic acid. The preparation, number, and type of samples required may vary locally, and samples are often performed by laboratory staff; however, basic requirements generally include smears (6 slides) and squash slides (≥ 2 slides), which are then air dried and methanol fixed (Lee et al., 2008):
  - Smears are prepared by placing a drop of aspirate on a glass slide using a glass spreader. The spreader is placed in front of the drop of aspirate at an angle of approximately 30° and pulled back to make contact with the drop. The spreader is then pushed forward in a smooth action, in contact with the slide.
  - Squash slides are prepared by placing a drop of bone marrow containing particles in the middle of one slide and then placing a second slide on top of the first without any additional pressure. The slides are drawn apart away from each other, in the direction of the long axis of the slide.
10. Attach a second syringe to the aspiration needle to obtain further samples for supplementary tests such as cytogenetic studies (samples are generally placed into preservative-free heparin or sterile tissue culture media, depending on local requirements) and molecular genetic studies (samples are generally placed into an anticoagulant). These samples should be handled as specified by local laboratories.

**Biopsy**

Bone marrow biopsies are obtained using specialized, two-part needles such as the Jamshidi or Islam needle. To perform bone marrow biopsies, practitioners should:

1. Make an incision at the puncture site with a scalpel blade. If the bone marrow aspirate was performed first, use the same incision but adjust the angle slightly so the biopsy needle enters the bone at a different position, 0.5 to 1 cm away from the aspiration needle (Lee et al., 2008; Malempati et al., 2009).
2. Using gentle but firm pressure, advance the needle while rotating it in an alternating semicircular clockwise–counterclockwise motion. In general, during a posterior iliac crest biopsy, the needle should be advanced at an angle completely perpendicular to the bony prominence of the iliac crest (Malempati et al., 2009). Once the needle passes through the cortical bone and enters the marrow cavity, there is generally a decrease in resistance, and the needle should stay in place without being held.
3. Remove the stylet from the middle of the needle, and advance the empty needle to a depth of approximately 2 cm into the marrow using a clockwise twisting motion. The stylet can be gently introduced back into the needle unit until resistance is met to help assess the specimen depth (Malempati et al., 2009).
4. Rotate the needle with quick, full twists several times to the right and to the left, and rock it gently to fully separate the bone marrow biopsy specimen.
5. Slowly remove the needle.
6. After withdrawing the needle, place gauze over the incision site, and use a thinner sterile stylus or probe to push the bone marrow biopsy gently out of the needle onto sterile gauze.
7. The sample should be at least 1.5 to 2 cm in length. If the sample is inadequate, the procedure should be repeated.
8. For touch-preparation slides, take a glass slide and gently touch the slide to the biopsy at three to four sites on the slide.
9. Put the biopsy sample either in a sterile vial or into fixative, according to local laboratory instructions.

**Post Procedure**

After performing bone marrow aspiration or biopsy, practitioners should:

1. Apply pressure with the thumb or fingers to the procedure site for approximately 5 minutes until bleeding has stopped.
2. Remove drapes and completely remove any povidone–iodine from the skin with alcohol swabs or warm water to avoid potential itching or an allergic response.
3. Affix clean or antibiotic-soaked gauze pads over the procedure site using pressure tape.
4. If possible, encourage the patient to lie with his or her weight on the incision site to assist hemostasis for an additional 15 to 30 minutes.
5. Advise the patient to remove the dressing the following day and to avoid bathing or swimming for 48 hours after the procedure. Advise the patient to contact his or her physician if pain persists for more than 24 hours or if bleeding is noted at the procedure site during the next few days. Warn the patient to contact his or her physician immediately if he or she experiences a temperature above 101°F (38.3°C) or evidence of infection (e.g., inflammation and pus at the biopsy site); warn the patient to be alert to redness or swelling, which may occur prior to pus.
6. Advise the patient when the results are likely to be available.
7. Manage postprocedural pain with acetaminophen.

**Potential Complications**

Adverse events following bone marrow biopsy are generally rare (Bain, 2006; Sarigianni et al., 2011) but can have a considerable effect on patients. Hemorrhage is the most common and most serious event, especially in patients with thrombocytopenia and in those on aspirin, warfarin, or heparin (Bain, 2006). Patients at risk of bleeding due to thrombocytopenia, aspirin use, or anticoagulation should be evaluated and, if necessary, treated prior to the procedure to reduce the risk of bleeding. Other potential complications include persistent pain, fracture (linked to osteoporosis), trauma to surrounding structures (such as damage to arteries), and infection, although they are rarely reported (Bain, 2006; Malempati et al., 2009).

## CONCLUSIONS

Examination of bone marrow using bone marrow aspirate and bone marrow biopsy is essential for the accurate diagnosis and risk stratification of patients with suspected MDS. Knowledge of what constitutes an adequate sample and proper bone marrow sampling procedure can help advanced practitioners obtain quality samples while minimizing patient discomfort and risk. Awareness and mitigation of potential complications combined with proper postprocedural care can also optimize patient outcome. Patients have different experiences with the procedure, including the level of pain encountered. Open communication prior to and during the procedure is key to improving the patient’s experience.

Increasingly, advanced practitioners are being called upon to perform invasive procedures such as bone marrow biopsies and aspirates. Advanced practitioners can play a central role in the development of procedural guidelines, structured training, and assessment programs to ensure both the quality of the samples obtained and patient safety and satisfaction with the procedure (Jackson, Guinigundo, & Waterhouse, 2012).

**Acknowledgments**

Editorial and writing support in the preparation of this manuscript was provided by Keisha Peters, MSc, of Excerpta Medica, and funded by Celgene Corporation. The authors are fully responsible for content and editorial decisions for this manuscript. The authors also wish to thank the MDS Foundation International Nurse Leadership Board.
